# Diabetes and hyperglucosuria exacerbate the severity of urinary tract infection caused by uropathogenic *E*. *coli* in the mouse model

**DOI:** 10.1128/iai.00172-26

**Published:** 2026-04-30

**Authors:** Sarita Neupane, Iris George, Veerakit Vanitshavit, Tessa Hejtmancik, Da Mi Kim, Gus Wright, L. Garry Adams, Sargurunathan Subashchandrabose

**Affiliations:** 1 Department of Veterinary Pathobiology, College of Veterinary Medicine and Biomedical Sciences, Texas A&M University 14736, College Station, Texas, USA; University of California San Diego School of Medicine, La Jolla, California, USA

**Keywords:** diabetes, T2DM, UTI, UPEC, dapagliflozin, T2DM mouse model, cystitis, pyelonephritis

## Abstract

Diabetes and urinary tract infection (UTI) are major global public health problems. We investigated the effects of glucose on bacterial colonization and host response to UTI caused by uropathogenic *Escherichia coli* (UPEC) in a mouse model of type 2 diabetes mellitus (T2DM) of polygenic origin. Dapagliflozin was used to induce hyperglucosuria in diabetic and healthy adult mice before and during experimental UTI with UPEC. Glucose, bacterial load, cytokines, tissue damage scores, and myeloid cell composition during acute UTI were determined. A UPEC mutant lacking a gluconeogenesis pathway was constructed to test the role of glucose on pathogen fitness in human urine and during UTI in diabetic mice. A significant increase in UPEC load was observed in the urinary tract of hyperglucosuric diabetic mice with a higher incidence of systemic dissemination and a higher degree of cystitis and bladder neutrophil counts compared to vehicle-treated controls. We also observed higher bacterial titer in vehicle-treated diabetic mice compared to healthy mice. While there was a significant increase in tissue damage in diabetic, hyperglucosuric mice, the levels of TNF-α, IL-1β, and IL-6 in the urinary tract were similar across treatment groups and genotypes. Our *in vitro* and *in vivo* competition experiments highlight the importance of the gluconeogenesis pathway in UPEC for optimal fitness of the pathogen in both healthy and diabetic hosts. Collectively, our results indicate that both hyperglucosuria and T2DM promote the onset of severe UTI, and systemic dissemination of urinary tract origin is likely caused by altered neutrophil trafficking to the urinary bladder in a mouse model.

## INTRODUCTION

Urinary tract infection (UTI) is one of the most common infectious illnesses globally, affecting ~400 million people each year (1–3). Urinary bladder infection, resulting in inflammation, pain, and discomfort, is the most common manifestation of UTI (4). Although less common, untreated UTI can lead to serious complications, such as pyelonephritis, bacteremia, urosepsis, and death (2, 4, 5). Uropathogenic *Escherichia coli* (UPEC) causes 75%–95% of uncomplicated UTIs in both healthy and diabetic individuals (1, 6, 7). In the US, diabetes affects ~10%, with ~40% at risk (prediabetic) in 2021, respectively (8). Type 2 diabetes mellitus (T2DM) is the predominant clinical presentation in 90%–95% of patients with diabetes (8). Patients with diabetes are a high-risk group for UTI associated with serious complications (9–13). Although the epidemiological link between UTI and diabetes is well-established, the underlying mechanisms are not completely understood.

Glucosuria has been suggested to increase UTI and its complications in patients with diabetes (12). In mice with T1DM, neutrophil mobilization and IL-6 production were subdued during UTI (14, 15). Signaling via insulin receptor promotes urothelial integrity and antibacterial activity in the bladder and kidneys of healthy mice with UTI, compared to *ob/ob* mice with T2DM (16–18). Hyperglucosuria increases bacterial colonization and UTI severity in healthy mice (19). Furthermore, glucosuria affects the virulence of diverse uropathogens, including UPEC (20–22). However, the role of glucosuria in UTI in a mouse model of T2DM of polygenic origin that closely emulates the pathobiology of T2DM in people remains to be elucidated.

Gliflozins used in the treatment of T2DM and some renal diseases inhibit sodium-glucose cotransporter 2 (SGLT2) to prevent renal glucose reabsorption, resulting in hyperglucosuria and normoglycemia (23–26). Clinical data suggest that gliflozins pose minimal-to-no risk for bacterial UTI and increased risk for urogenital fungal infection (27–33). Here, we use dapagliflozin as a pharmacological tool to dissect the role of hyperglucosuria in UTI severity in T2DM (19, 34, 35). We hypothesized that dapagliflozin-induced hyperglucosuria exacerbates the severity of bacterial UTI in the KK-Ay mouse model of T2DM of polygenic origin mice. Our findings demonstrate the critical role of urinary glucose in exacerbating UTI outcomes in diabetic hosts.

## MATERIALS AND METHODS

### Bacterial strains, mutants, and culture conditions

Clinical UPEC strain UTI89 (36) and its isogenic ∆*pckA* (chloramphenicol-resistant) mutant, constructed using Lambda-red recombineering (37), were used in this study. Strains were cultured in lysogeny broth (LB: 10 g/L peptone, 5 g/L yeast extract, 5 g/L sodium chloride, and +/− 15 g/L agar) with or without chloramphenicol at 37°C.

Oligonucleotide primers used for screening and mutating *pckA* were 5′GCATCCGGGCAGTAGTATTT3′, 5′GGATAACCATAACGGGATGC3′, 5′GGAGTTTTTTGTCAAATATGAATTTCTCCAGATACGTAAATCTATGAGCC3′, and 5′CCGTTTTGCTTTCTATAAGATACTGGATAGATATTCTCCAGCTTCAAATC3′, respectively. Pooled filter-sterilized human urine (Cone Bioproducts) from five adult female donors without a history of UTI/diabetes was used as described in a previous study (38). Aliquots of urine samples were stored at −80°C. Freshly thawed samples were used for bacterial culture. Urine pH and specific gravity were measured for each aliquot to ensure consistency over time. There was no detectable glucose in these human urine samples. Bacteria were cultured in LB or human urine (+/− glucose) at a 1:1,000 dilution, and OD_600_ was measured hourly (Cytation 5, Agilent Biotek) (38).

For *in vitro* competition, UPEC wild-type and *∆pckA* were mixed in a 1:1 ratio, diluted 1,000-fold in indicated media and incubated overnight. CFUs were enumerated by plating on LB agar +/− chloramphenicol at T0 and T24 h. The competitive index was calculated as the ratio of mutant to wild-type at T24 and normalized to the ratio of mutant to wild-type at T0 (39, 40).

Biofilm assay was performed as previously described (41, 42). Briefly, UPEC strains were cultured for 20 h at 37°C and 30°C in 96-well polystyrene plates statically. OD_600_ was measured; biofilms were washed, stained with 0.1% crystal violet, dried, and dissolved in 30% acetic acid; OD_550_ was recorded; and biofilm was normalized to the growth (OD_550_/OD_600_).

### T2DM mouse model of UTI

Experiments were approved by Texas A&M University (IACUC approval #2024-0201). Six to 8-week-old female yellow KK.Cg-A^y^/J (KK-Ay) mice (#002468, Jackson Laboratory) were used to model T2DM of polygenic origin (43–46). Age- and sex-matched black wild-type littermates were used as healthy controls. Mice were randomized to groups normalized for body weight within each genotype. Mice were fasted for 4 h prior to determining blood glucose levels. Dapagliflozin (10 mg/kg/day, Advanced ChemBlocks) was gavaged at the indicated days between 8 and 10 AM immediately after urine collection (19). Controls received vehicle (0.5% wt/vol carboxymethylcellulose in water). Urine was collected by scruffing and holding the mice over a sterile surface for ~20 s to collect voided urine and was consistently performed between 8 and 10 AM to avoid diurnal changes in composition. Void spot assays were performed to evaluate the micturition pattern (47). Mice (*N* = 5−10/group) were transurethrally inoculated with 10^8^ CFU of UPEC alone or with *∆pckA* (1:1) ratio in 50 µL of PBS using a syringe pump, as previously described (19). Mice were euthanized 48 h post-infection. The entire abdominal and inguinal fat pads were dissected and weighed. Samples were collected aseptically, homogenized, diluted, and plated to determine bacterial load. Bacterial plating and CFU counting were performed by operators blinded to the sample origin. Experiments were repeated at least thrice independently for determining bacterial burden, histopathology, and flow cytometry outcomes.

### Histopathology

Urinary bladders and kidneys were fixed in formalin, embedded in paraffin, sectioned, and stained with hematoxylin and eosin. Histopathology scores for inflammation were determined in a blinded manner by board-certified Veterinary Anatomic Pathologists using the criteria listed in Box 1, as described previously (19, 48). Scores for each criterion were added to generate the total tissue damage score for a given sample.

Box 1. **Histopathology scoring criteria**

**Cystitis**

Luminal bacteria, luminal/adherent biofilm-like clusters of bacteria in the lumen, adherent bacteria, intraepithelial bacteria, luminal/intraepithelial neutrophils, intraepithelial pustules, submucosal edema, and the following urothelial changes: ulceration and sloughing, thickening and remodeling, mitotic figures, and ballooning degeneration.

**Pyelonephritis (pelvis or parenchyma)**

Neutrophils, edema, bacteria, necrosis, ballooning degeneration, microabscess, mitoses, thickening and remodeling, mineralization, and unilateral or bilateral renal involvement.

0, no change; 1, minimal change; 2, moderate change; 3, marked change; and 4, extensive change in bladders, pelvis, or parenchyma.

### Diabetic Bio-Plex assay and urine and serum chemistry quantification

Blood was collected via cardiac puncture post-mortem. The serum was used in a Bio-Plex Pro mouse Diabetes 8-Plex Assay (Bio-Rad #171F7001M) to quantify ghrelin, insulin, leptin, PAI-1, resistin, GIP, GLP-1, and glucagon. Assays were performed in duplicate, and the concentration was calculated using standard curves. Urinary glucose and creatinine were determined by colorimetric assays (Cayman Chemicals), and blood glucose levels at indicated times were determined using a glucometer (Bayer) (19). Urinary sodium and urea nitrogen, and serum glucose, creatinine, cholesterol, HDL, LDL, triglycerides, and urea nitrogen were determined using DXC 700 AU Chemistry Analyzer (Beckman Coulter).

### Flow cytometry, CBC, and ELISA

Neutrophils and macrophages were detected using these antibodies (Biolegend: PerCP anti-mouse CD45 antibody, 103129; Alexa Fluor 488 anti-mouse CD11b antibody, 101219; BV711 anti-mouse Ly-6G antibody, 127643; Spark YG 570 anti-mouse F4/80 antibody, 123159; Alexa Fluor 647 anti-mouse CD11c antibody, 117314; and PE-Cyanine7 anti-mouse CD163 antibody, 155319) by operators blinded to sample origin. Bladder sections were digested with collagenase IV and DNase I, filtered (Thermofisher, #00-8222-49), stained with live/dead marker (Zombie NIR, Biolegend #423105), blocked with Fc block (BD Biosciences, #553141), stained with antibody cocktail, and fixed (eBioscience #00-8222-49). Cells were resuspended in 200 µL FACS buffer (PBS+2% FPBS) for use in the Cytek Aurora Spectral Flow Cytometer and analyzed with FlowJo version 10 with the gating strategy depicted in Fig. S3. CBC was performed using a Zoetis VetScan HM5 system. Assays were performed at least in duplicate. Urine and clarified tissue homogenates (bladder and kidneys) were used for ELISA to detect myeloperoxidase (MPO, ThermoScientific), TNFα, IL-1β, and IL-6 (R&D systems), as previously described (48).

### Statistical analysis

Data were analyzed using GraphPad Prism with statistical tests, including post-hoc multiple comparisons test, as indicated in figure legends. Normality of data was assessed using GraphPad Prism, and appropriate parametric or non-parametric tests were applied based on the number of groups compared. Each symbol in a figure corresponds to data collected from a mouse. *P* < 0.05 was considered a statistically significant difference.

## RESULTS

### Dapagliflozin induced hyperglucosuria in diabetic and healthy mice

**Fig 1:**
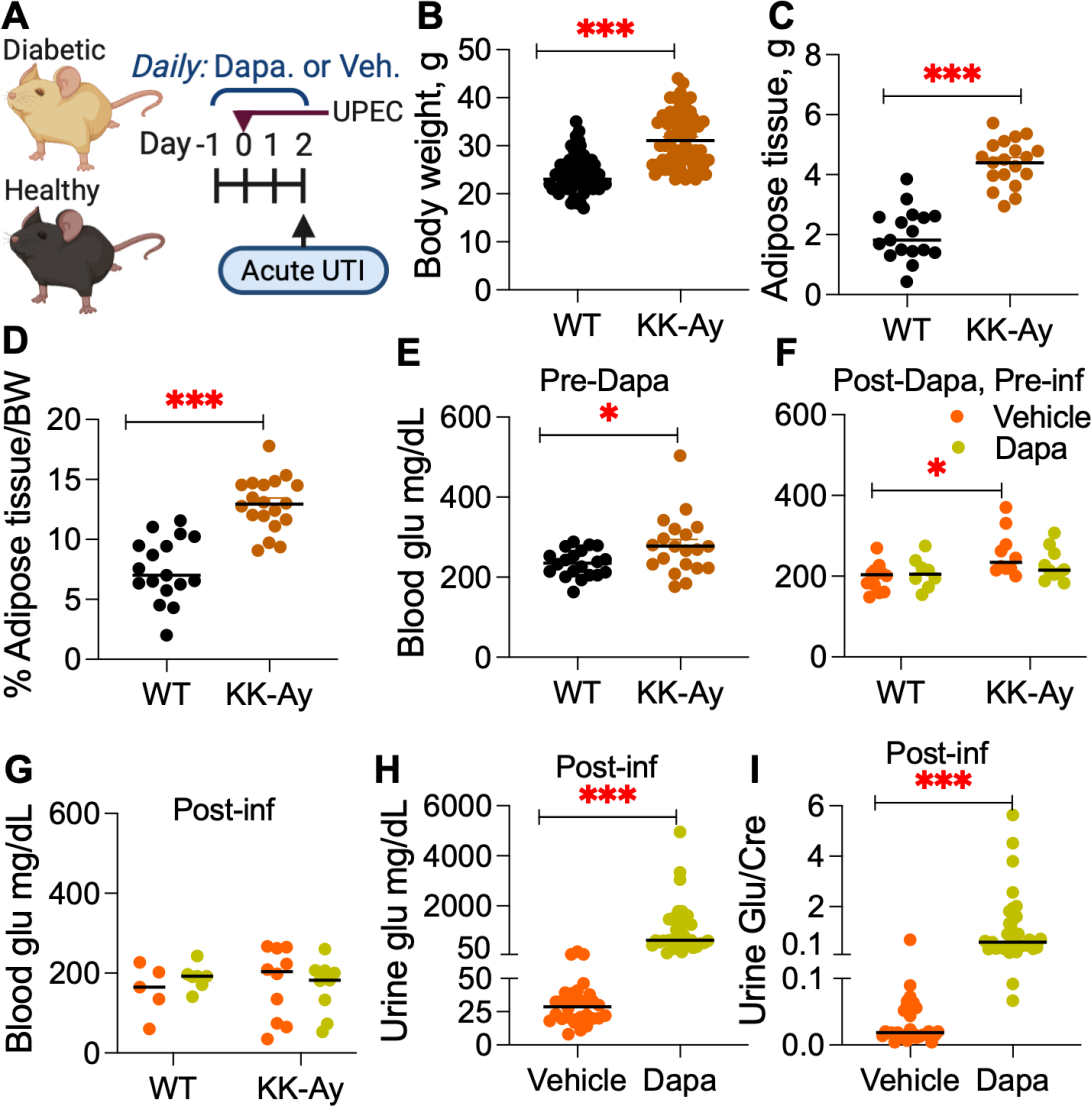
Timeline for UTI and induction of hyperglucosuria in a T2DM mouse model. Adult female diabetic KK-Ay mice and healthy WT littermates (*n* = 5–10/group/experiment) gavaged with vehicle (veh.) or dapagliflozin (dapa.) group and inoculated with UPEC strain in the bladder and euthanized during acute UTI (**A**). Body weight, absolute and normalized inguinal and abdominal fat pad weight (**B–D**). Fasting blood glucose levels were measured before the study (pre-dapa), after gavage and before infection (post-dapa, pre-inf), and on day 2 (post-inf) (**E–G**). Absolute and normalized urine glucose on day 2 was determined (**H and I**). Each symbol represents a mouse, and bars indicate the median. *P < 0.05, **P < 0.01, ***P < 0.001, *t*-test.

Adult, female, diabetic (KK-Ay), and healthy (WT-littermate controls) mice were administered dapagliflozin or vehicle (Fig. 1A). The primary outcome for this experiment was to determine the impact of dapagliflozin in inducing hyperglucosuria, and if there was a difference in this response between healthy and diabetic mice. Diabetic mice were heavier than controls, with larger abdominal and inguinal fat pads (Fig. 1B through D). Urine voiding pattern, determined by void spot assays, was similar between healthy and diabetic mice (Fig. S1A and B). As expected, fasting blood glucose was significantly higher in diabetic mice compared to controls (Fig. 1E). Blood glucose was lower in the dapagliflozin-treated group, compared to pre-treatment (Fig. 1F). Post-infection, fasting blood glucose was similar across groups (Fig. 1G). Dapagliflozin-treated mice had significantly higher glucosuria compared to vehicle controls (Fig. 1H and I). Since control mice weighed less and had less abdominal and inguinal fat pads than the diabetic mice (Fig. 1B through D), and the known effect of dapagliflozin on increasing renal sodium excretion, we determined nitrogen and sodium levels in urine. Urine urea nitrogen (UUN) was significantly reduced in dapagliflozin-treated healthy mice compared to vehicle-treated controls (Fig. S1C and D), consistent with its known renal effect (25, 26). Diabetic and control mice had a ~2-fold lower UUN during dapagliflozin treatment. Urine sodium level was similar across groups (Fig. S1E and F).

### Hyperglucosuric diabetic mice were highly colonized by UPEC during UTI with increased systemic dissemination

**Fig 2:**
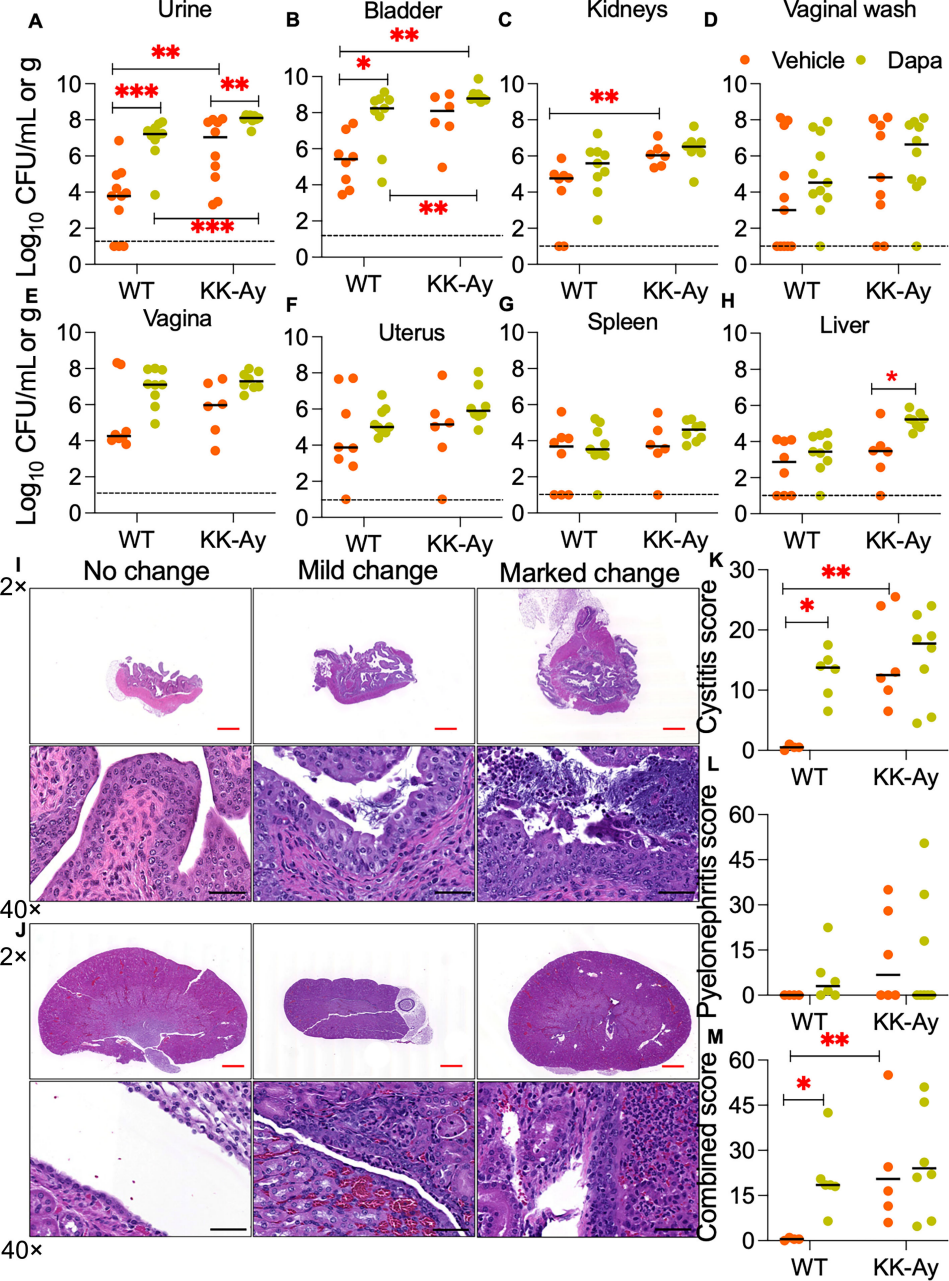
UPEC colonization in the urogenital tract, systemic dissemination, and tissue damage during UTI. Female diabetic and healthy WT littermates were inoculated with UPEC strain UTI89, and bacterial load was determined at 48 h post-inoculation in urine (**A**), urinary bladder (**B**), kidneys (**C**), vaginal lavage (**D**), vagina (**E**), uterus (**F**), spleen (**G**), and liver (**H**). Representative hematoxylin and eosin-stained bladder (**I**) and kidney (**J**) sections. Cystitis, pyelonephritis, and combined score from all mice (**K–M**). Each symbol represents a mouse, and the bars indicate the median. Red scale bar, 1 mm. Black scale bar, 50 µm. Dapa, dapagliflozin. *P < 0.05, **P < 0.01, ***P < 0.001. Mann-Whitney test.

The primary outcome for this experiment was to determine the impact of dapagliflozin on altering bacterial burden during experimental UTI, and if there was a difference in this response between healthy and diabetic mice. Dapagliflozin-treated diabetic and healthy mice had significantly higher bacterial burden in urine compared to vehicle-treated controls (Fig. 2A). Vehicle-treated diabetic mice also had significantly higher bacterial load in urine compared to vehicle-treated healthy mice. Bladder and kidney bacterial titers were significantly higher in the diabetic group compared to healthy controls with vehicle treatment (Fig. 2B and C). The effect of dapagliflozin was not statistically significant within diabetic groups, except in urine. However, a significant increase in bacterial load was observed in dapagliflozin-treated healthy mice compared to vehicle-treated groups (Fig. 2A through C). Median *E. coli* load was higher, albeit not significant, between diabetic and healthy groups, and between dapagliflozin- and vehicle-treatment groups in the reproductive organs (Fig. 2D through F). Urine and serum glucose levels correlated weakly with the urine bacterial load, with better correlation in diabetic mice than in controls (Fig. S1G and H). Bacterial colonization in the spleen and liver is a marker of bacteremia and systemic dissemination in mice, like UTI complications in people (41, 49, 50). During acute UTI, 100% of dapagliflozin-treated diabetic mice had UPEC infection in spleen and liver compared to 60%–80% in other groups (Fig. 2G and H). Bacterial burden in the livers of dapagliflozin-treated diabetic mice was statistically significantly higher compared to vehicle-treated controls.

### Cystitis and pyelonephritis were more frequent and severe in diabetic mice with UTI

Representative images depict the range of inflammatory changes for cystitis and pyelonephritis (Fig. 2I and J; Fig. S2C through H). Inflammation score criteria for cystitis and pyelonephritis are presented in Box 1. Diabetic mice had significantly higher cystitis scores compared to controls (Fig. 2K). Dapagliflozin-treated healthy mice had significantly higher cystitis scores than vehicle. Within the diabetic group, dapagliflozin-treated mice trended toward higher cystitis scores. We observed no bladder lesions in the vehicle-treated, healthy, WT mice. More diabetic mice had a severe pyelonephritis and combined score than healthy mice (Fig. 2L and M).

The urinary bladders contained luminal UPEC surrounded by neutrophils and sloughed, degenerated umbrella cells, intraepithelial UPEC, transmigrating neutrophils, and aggregates, with ulceration, degenerative changes, and vacuolation (Fig. 2I; Fig. S2A through C). Submucosal edema, neutrophil infiltration, and reactive endothelial cells were also observed (Fig. 2I; Fig. S2A through C). These features were prominent in diabetic mice compared to healthy controls and further increased in dapagliflozin-treated diabetic mice (Fig. 2I; Fig. S2A through C). Overall, 97% (23/24) of mice developed various severities of urinary bladder lesions. Among them, 61% (14/23) were diabetic (dapagliflozin = 8, vehicle = 6), and 39% (9/23) were healthy, WT (dapagliflozin = 6, vehicle = 3).

Multifocal neutrophil infiltration in the renal pelvis, papilla, and inner medulla, with edema in the pelvis, was observed (Fig. 2J; Fig. S2B, D through F). Pelvic urothelium displayed swelling and vacuolation, neutrophil transmigration and intraurothelial microabscesses, necrosis, ulceration, hyperplasia, and karyomegaly (Fig. 2J; Fig. S2B, D through F). Medullary early abscesses were surrounded by neutrophils, fibroblasts, and few macrophages, and rare lymphocytes were also observed; 42% (10/24) of mice developed renal lesions, with unilateral and bilateral lesions in seven and three mice, respectively. Four mice (KK-Ay, dapagliflozin-treated = 1; KK-Ay, vehicle-treated = 2; WT, dapagliflozin-treated=1) had lesions only on left kidneys; three healthy, dapagliflozin-treated mice had lesions only on right kidneys; and three mice (KK-Ay, dapagliflozin-treated = 2; KK-Ay, vehicle-treated = 1) had bilateral renal lesions. All mice with renal lesions had ureteritis (Fig. S2H), pyelitis, and pyelonephritis.

### Dapagliflozin-treated diabetic mice exhibited higher bladder neutrophil infiltration

**Fig 3:**
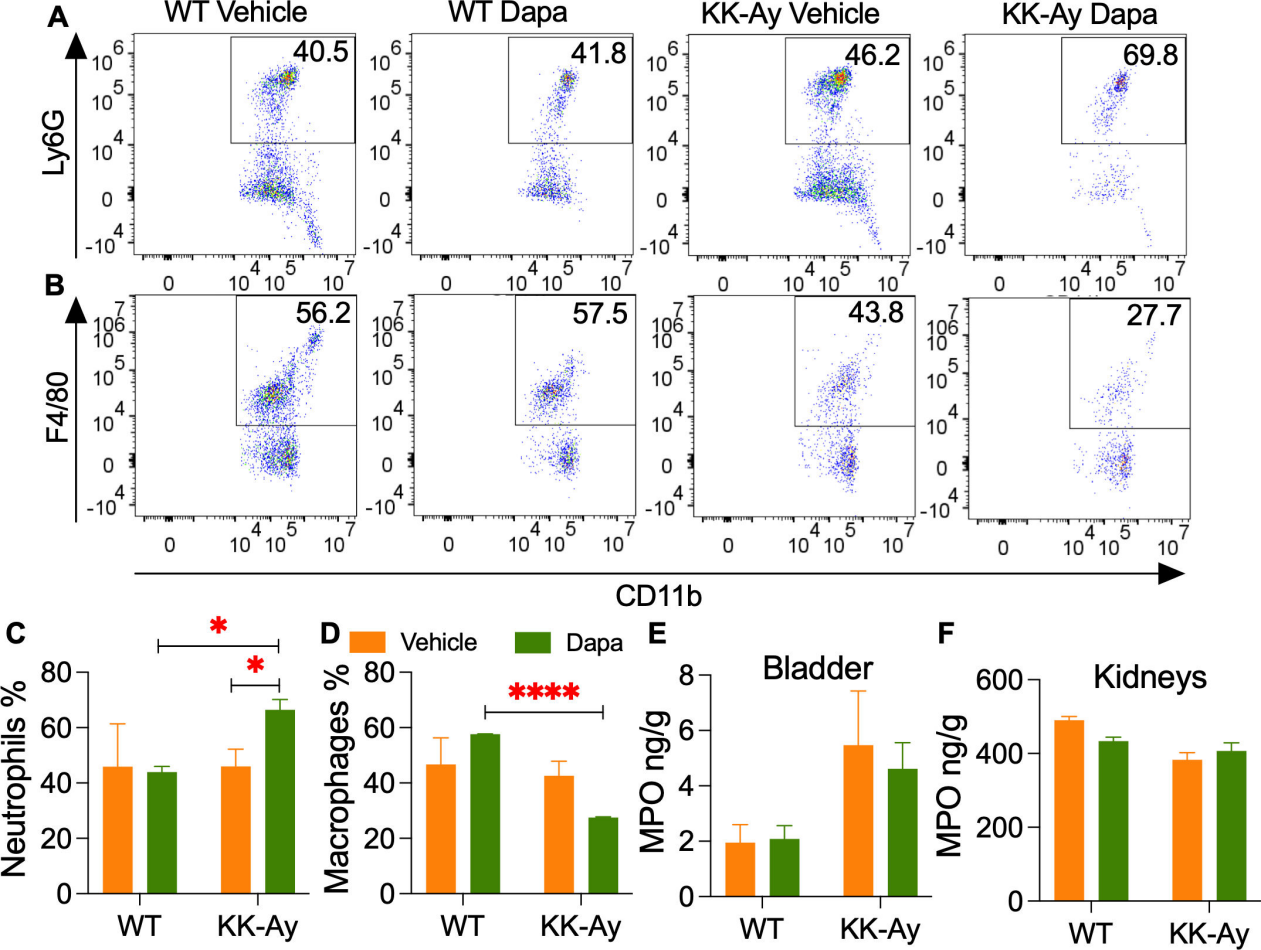
Neutrophil and macrophage infiltration in the urinary bladder during UTI. Urinary bladders from diabetic (KK-Ay) and healthy (WT) mice (*n* = 2–8/group) with UPEC-induced UTI were analyzed by flow cytometry. Representative images from counts for neutrophils (CD11b^+^Ly6C^+^) and macrophages (CD11b^+^F4/80^+^) (**A and B**). Neutrophils and macrophages as a percent of myeloid cells are presented in panels **C** and **D**. Myeloperoxidase (MPO) levels in the bladders and kidneys were quantified by ELISA in panels **E** and **F**. Mean + SEM. *P < 0.05, ***P < 0.001, *t*-test.

Representative plots of bladders from flow cytometry using a gating strategy and FMO controls (Fig. S3) are depicted in Fig. 3A and B. Dapagliflozin-treated diabetic mice had a significantly higher proportion of neutrophils (CD45^+^CD11b^+^Ly6G^+^) and decreased macrophages (CD45^+^CD11b^+^F4/80^+^) among myeloid cells compared to diabetic controls and dapagliflozin-treated healthy mice (Fig. 3C and D). M1 macrophages (F4/80^+^CD11c^+^) were higher in diabetic mice compared to healthy mice (Fig. S4A). M2 macrophages (F4/80^+^CD163^+^) were similar between treatment groups and genotypes (Fig. S4B); ~100% of urine myeloid cells were neutrophils, with rare macrophages (<1%), in dapagliflozin- and vehicle-treated diabetic mice (Fig. S4C and D). Blood counts of leukocytes, including neutrophils and macrophages, were similar across all groups (Fig. S5A through E). Bladder MPO levels were higher, but not significantly different, in diabetic mice compared to healthy mice (Fig. 3E). Kidney MPO levels and cytokines (IL1-β, IL-6, and TNF-α) were comparable across groups and genotypes (Fig. 3F; Fig. S5F through N).

### Diabetic mice had higher serum insulin post-infection compared to healthy mice

To get an insight into the hormonal, metabolite, and inflammatory changes associated with diabetes and bacterial UTI, we performed a Bio-Plex Pro mouse diabetes 8-Plex assay to quantify multiple biomarkers for diabetes in the serum. Insulin was ~2-fold higher in diabetic mice, compared to healthy mice (Fig. S6A). After dapagliflozin treatment, ghrelin was increased approximately 4-fold in diabetic mice, whereas only 2-fold was increased in healthy mice (Fig. S6B). Leptin, PAI-1, resistin, glucose, cholesterol, triglycerides, HDL, LDL, BUN, and creatinine were similar across treatment and genotypes (Fig. S6C through E and S7A through G). GIP, GLP-1, and glucagon were not detectable.

### Gluconeogenesis was required for the optimal fitness of UPEC during UTI

**Fig 4:**
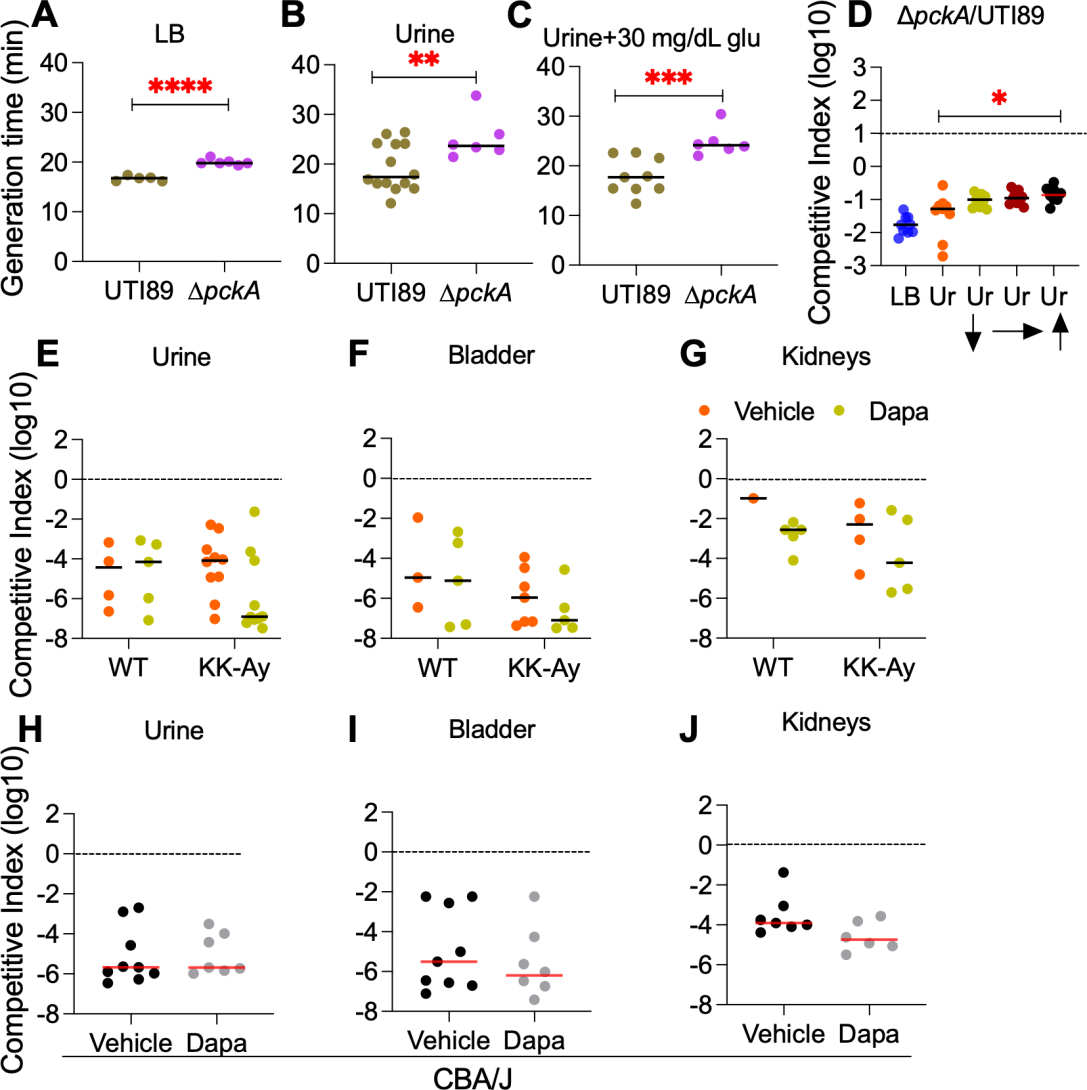
Role of gluconeogenesis in promoting *in vitro* and *in vivo* fitness of UPEC. Generation times of UPEC WT strain UTI89 and gluconeogenesis-defective *∆pckA* mutant LB (**A**), human urine (**B**), and urine with 30 mg/dL glucose to mimic glucosuria (**C**). *In vitro* competitive indices in LB, human urine, and urine with 30 mg/dL glucose (Ur↓), 300 mg/dL (Ur→), and 2,000 mg/dL (Ur↑) (**D**). *In vivo* competitive indices for urine, bladder, and kidneys were calculated from bacterial load from co-infection with UPEC UTI89 and *∆pckA* mutant in diabetic KK-Ay and control mice (**E–G**) or in non-diabetic CBA/J mice (**H–J**). Dotted line, no loss of fitness in mutant relative to parental wild-type strain (competitive index of 1). Each symbol represents a replicate or mouse, and bars indicate the median. Dotted line, no loss of fitness in the mutant relative to wild-type strain (competitive index = 1). *P < 0.05, **P < 0.01, ***P < 0.001, *t*-test.

We constructed a Δ*pckA* mutant to probe the role of central carbon metabolism in UPEC fitness (Fig. S8A). This mutant is incapable of generating glucose by gluconeogenesis from the TCA cycle (51). Wild-type and Δ*pckA* were cultured in LB and human urine with 0–2,000 mg/dL glucose mimicking hyperglycosuria (Fig. S8B through L). UTI89 had significantly shorter generation time, calculated using R studio (38), and higher final cell density compared to *∆pckA* (Fig. 4A through C; Fig. S8E through C). In human urine, the Δ*pckA* mutant formed significantly higher biofilm compared to LB (Fig. S8M and N). Co-culture experiments with UTI89 and *∆pckA* in LB and human urine with and without glucose revealed a severe fitness defect for Δ*pckA* (Fig. 4D). Glucose promoted, but did not restore, the wild-type level of fitness for Δ*pckA*.

To determine pathogen fitness in the diabetic urinary tract, we performed co-infection with WT UPEC strains UTI89 and UTI89*∆pckA* in healthy and diabetic mice treated with dapagliflozin and vehicle. Our results demonstrated a severe fitness defect in *∆pckA* in the urogenital tract of T2DM mouse model regardless of hyperglucosuria or diabetes (Fig. 4E through G; Fig. S8O through S). Next, we tested if failure of mutant colonization is specific to the diabetic mouse model. Co-infection with UTI89 and Δ*pckA* in non-diabetic CBA/J showed that the mutant exhibited poor fitness during UTI (Fig. 4H through J; Fig. S8T through Y). Collectively, our results indicate that gluconeogenesis is indispensable for optimal colonization of UPEC in healthy and diabetic urinary tracts.

## DISCUSSION

Millions of people develop diabetes worldwide, with about 90%–95% being T2DM (8). Diabetes mellitus is well known to exacerbate the risk for the development of UTI (52). The predominant etiological agent of UTI in both diabetic and non-diabetic populations is UPEC (2, 6, 53). Whether diabetes increases UTI susceptibility by glucose or diabetes-associated metabolites-fueled bacterial growth, changes in host response to pathogens, or a combination of both is still unclear (14, 15, 18, 19, 54). Gliflozins are SGLT2 inhibitors commonly used for glycemic management, and whether their use increases UTI risk is an important question in UTI/diabetic fields (32, 33, 35). Here, we used dapagliflozin to investigate the role of glucose to determine the impact of glucosuria on the outcomes of bacterial UTI in the polygenic origin of a T2DM mouse model that more closely emulates the pathophysiology of diabetes in humans. We have previously demonstrated that dapagliflozin-induced glucosuric, female, non-diabetic, CBA/J mice had higher bacterial colonization in the urinary tract during acute and chronic UTI with development of cystitis, pyelonephritis, and systemic dissemination at a higher frequency than controls (19). As the next important step toward understanding the effects of SGLT2 inhibitors on UTI, here, we report that both hyperglucosuria and T2DM exacerbate the severity of bacterial UTI in a mouse model and that the gluconeogenesis pathway is critical for UPEC to successfully colonize both diabetic and non-diabetic hosts.

Our results demonstrate that dapagliflozin-induced hyperglycosuria in the presence of diabetes-associated pathological changes increases UPEC load in the urinary tract and systemic dissemination of urinary tract origin in female diabetic mice with UTI. This suggests an impaired ability to contain and resolve UPEC colonization under hyperglucosuric conditions in diabetes, likely driven in part by drug-induced glucosuria. Additionally, significantly increased bacterial titer in urine, bladder, and kidneys in vehicle-treated diabetic mice compared to vehicle-treated healthy mice is consistent with diabetes as a risk factor for UTI, as previously reported (9, 13). Alteri et al. demonstrated that gluconeogenesis is essential for UPEC fitness in the urinary tract of non-diabetic mice (51, 55). Since diabetic urine is glucose-rich, it has been speculated that gluconeogenesis may not be involved in pathogen fitness in diabetic hosts. Our *in vitro* experiments reveal that glucose can partially enhance the growth defect of the gluconeogenesis-deficient human urine *∆pckA* mutant. If urine glucose fueled microbial growth, then the *∆pckA* mutant would not be expected to exhibit a fitness defect in glucosuric hosts. However, our results indicate that the mutant has ~10,000-fold decreased fitness in the urine and bladders, suggesting that glucose availability is not the main driver of microbial growth in the urinary tract. In contrast, our results clearly indicate that gluconeogenesis is indispensable for UPEC fitness regardless of urine glucose content and is consistent with the preferential use of amino acids and peptides as carbon and nitrogen sources by UPEC (51, 55). Collectively, our findings suggest that diabetes exacerbates UTI severity by modulating the urinary microenvironment to favor bacterial growth while simultaneously dysregulating the immune function with excessive neutrophil but reduced macrophage presence, impairing effective bacterial clearance, tissue repair, and hemostasis. Since glucose availability does not directly support UPEC growth, based on our findings on the fitness of the *∆pckA* mutant in healthy and diabetic mice, it is important to investigate T2DM-dependent changes in other urinary metabolites, which might affect UPEC colonization. An important future direction for the work presented here is to investigate how glucose and other T2DM-dependent urinary metabolites affect urothelial biology and innate immune effectors, resulting in exacerbated UPEC colonization during UTI.

Our findings are consistent with previous reports where dapagliflozin increases bacterial and fungal load in urinary tract of non-diabetic mouse models (19, 34). Additionally, our study expands on the few previous reports on the effects of hyperglucosuria in the mouse model of UTI. Saenkham et al. reported higher bacterial burden in the urinary tract with more frequent development of systemic dissemination of UPEC of UTI origin in dapagliflozin-treated non-diabetic (CBA/J) mice (19). Dapagliflozin or canagliflozin-induced hyperglucosuria in non-diabetic (C57BL/6J) mice leads to increased urogenital infections with *Candida albicans* (34). Using a streptozotocin-induced T1DM mouse model, Rosen et al. have reported increased bacterial load and tissue damage in the urinary tract during experimental UTI (14). They observed severe, acute, suppurative, interstitial pyelonephritis in diabetic mice with large colonies of UPEC UTI89 within the lumen of renal tubules, mostly confined to the renal cortex and medullary regions. However, most of our hyperglucosuric, diabetic mice had neutrophilic pyelonephritis, and rarely, bacteria were seen on histopathology. Marked interstitial nephritis with myriad luminal bacterial colonies within tubules in a study by Rosen et al. may be partly due to diabetic-induced changes leading to compromised innate immunity in the streptozotocin-induced mouse model. Recently, Salamon et al. reported increased UPEC load in the urinary tract of dapagliflozin-treated non-diabetic mice (C3H/HeOuJ), whereas another non-diabetic (C57BL/6J) mouse had comparable bacterial load between dapagliflozin-treated and vehicle groups, and diabetic (*db*/*db*) mice developed reduced UPEC UTI89 burden at 7 days post-infection UTI (35). They also noted rare to minimal inflammation, mostly limited to the pelvis (pyelitis) in dapagliflozin-treated mice (35). They used diabetic, *db/db* mice, which develop diabetes of monogenic origin compared to the KK-Ay mice, in this study to model T2DM of polygenic origin to better emulate the pathophysiological changes in humans (43), compared to T2DM of monogenic origin in *db/db* mice. This study focuses on acute UTI, whereas Salamon et al. addressed chronic UTI outcomes. A recent report by Sendtner et al. revealed that empagliflozin, another SGLT2 inhibitor, does not increase UPEC strain 536 colonization in C57BL/6 mice with experimental pyelonephritis (56). They also demonstrate that there is no impairment of renal antibacterial defense during empagliflozin use (56). Subdued urinary neutrophil levels and IL-6 levels were observed in streptozotocin-induced diabetic mice with UTI (15), whereas proinflammatory markers, including IL-6, were similar in diabetic *versus* healthy mice, as well as in dapagliflozin *versus* vehicle-treated groups in our study. Differences in UTI outcomes in these reports could be due to genetic variations in mouse strains used to model UTI, mono/polygenic T2DM, T1/T2 DM, and acute *versus* chronic UTI endpoints.

A limitation of our study is the inability to generalize our results in males since we used only female mice. The influence of gender on UTI is complex, and male mice can develop even more serious complications than females due to anatomy, physiology, and hormonal influence that inhibit complete pathogen clearance once an infection is established, leading to pathogen reservoir formation and recurrent infection (57). Future investigations should test the impact of SGLT2 inhibitors on UTI outcomes in males. A prototypical, widely used UPEC strain UTI89 was used in our study. Future studies with other uropathogens are needed to rule out the possibility of UPEC-specific effects on colonization in hyperglucosuria condition in the T2DM model. We focused only on dapagliflozin because we have previously reported that dapagliflozin and canagliflozin exert similar effects on bacterial UTI in mice (19). Given the availability of several other FDA-approved SGLT2 inhibitors in clinical use, it is possible that other gliflozins may affect UTI differently due to variation in pharmacokinetics.

Overall, our study revealed that hyperglucosuria as well as diabetes-associated pathological changes lead to a higher risk of UTI in T2DM. Higher bacterial load in diabetic mice compared to healthy controls indicates a role for diabetes-associated changes, such as alteration in innate immunity and urinary metabolites, in fueling bacterial growth. These findings are consistent with clinical data of UTI in diabetes, where complications of UTI are more severe and difficult to manage (9–11). Findings reported here and in prior reports on SGLT2 inhibitors demonstrate that hyperglucosuria triggered by these drugs significantly exacerbates uropathogen colonization during experimental UTI. However, it is important to note that there is little-to-no association between SGLT2 inhibitor use and UTI in people with T2DM (30, 58). Therefore, this mouse model needs to be further refined, including determining a lower infectious dose that results in reproducible infection without overwhelming the host, to recapitulate the pathogenesis of UTI in people with T2DM taking SGLT2 inhibitors for glycemic management. In conclusion, both hyperglucosuria and diabetes-associated changes exacerbate UPEC colonization and UTI severity in the KK-Ay mouse model of T2DM.

## SUPPLEMENTAL MATERIAL

Supplemental material iai.00172-26-s0001.pdf Fig. S1 to S8.

ASM does not own the copyrights to Supplemental Material that may be linked to, or accessed through, an article. The authors have granted ASM a non-exclusive, world-wide license to publish the Supplemental Material files. Please contact the corresponding author directly for reuse.

## References

[1] Flores-Mireles AL, Walker JN, Caparon M, Hultgren SJ. 2015. Urinary tract infections: epidemiology, mechanisms of infection and treatment options. Nat Rev Microbiol 13:269–284. doi:10.1038/nrmicro3432 25853778 PMC4457377

[2] Foxman B. 2014. Urinary tract infection syndromes: occurrence, recurrence, bacteriology, risk factors, and disease burden. Infect Dis Clin North Am 28:1–13. doi:10.1016/j.idc.2013.09.003 24484571

[3] Stamm WE, Norrby SR. 2001. Urinary tract infections: disease panorama and challenges. J Infect Dis 183 Suppl 1:S1–4. doi:10.1086/318850 11171002

[4] Hooton TM. 2012. Clinical practice. uncomplicated urinary tract infection. N Engl J Med 366:1028–1037. doi:10.1056/NEJMcp1104429 22417256

[5] Dimitrijevic Z, Paunovic G, Tasic D, Mitic B, Basic D. 2021. Risk factors for urosepsis in chronic kidney disease patients with urinary tract infections. Sci Rep 11:14414. doi:10.1038/s41598-021-93912-3 34257397 PMC8277778

[6] Subashchandrabose S, Mobley HLT. 2015. Virulence and fitness determinants of uropathogenic Escherichia coli. Microbiol Spectr 3. doi:10.1128/microbiolspec.UTI-0015-2012 PMC4566162 26350328

[7] Hannan TJ, Totsika M, Mansfield KJ, Moore KH, Schembri MA, Hultgren SJ. 2012. Host-pathogen checkpoints and population bottlenecks in persistent and intracellular uropathogenic Escherichia coli bladder infection. FEMS Microbiol Rev 36:616–648. doi:10.1111/j.1574-6976.2012.00339.x 22404313 PMC3675774

[8] Anonymous. 2024. National diabetes statistics report website. Centers for Disease Control and Prevention. Available from: https://www.cdc.gov/diabetes/php/data-research/index.html

[9] Patterson JE, Andriole VT. 1997. Bacterial urinary tract infections in diabetes. Infect Dis Clin North Am 11:735–750. doi:10.1016/s0891-5520(05)70383-4 9378933

[10] MacFarlane IA, Brown RM, Smyth RW, Burdon DW, FitzGerald MG. 1986. Bacteraemia in diabetics. J Infect 12:213–219. doi:10.1016/s0163-4453(86)94112-5 3522748

[11] Muller LMAJ, Gorter KJ, Hak E, Goudzwaard WL, Schellevis FG, Hoepelman AIM, Rutten GEHM. 2005. Increased risk of common infections in patients with type 1 and type 2 diabetes mellitus. Clin Infect Dis 41:281–288. doi:10.1086/431587 16007521

[12] Geerlings SE. 2008. Urinary tract infections in patients with diabetes mellitus: epidemiology, pathogenesis and treatment. Int J Antimicrob Agents 31 Suppl 1:S54–7. doi:10.1016/j.ijantimicag.2007.07.042 18054467

[13] Paudel S, John PP, Poorbaghi SL, Randis TM, Kulkarni R. 2022. Systematic review of literature examining bacterial urinary tract infections in diabetes. J Diabetes Res 2022:3588297. doi:10.1155/2022/3588297 35620571 PMC9130015

[14] Rosen DA, Hung CS, Kline KA, Hultgren SJ. 2008. Streptozocin-induced diabetic mouse model of urinary tract infection. Infect Immun 76:4290–4298. doi:10.1128/IAI.00255-08 18644886 PMC2519435

[15] Ozer A, Altuntas CZ, Bicer F, Izgi K, Hultgren SJ, Liu G, Daneshgari F. 2015. Impaired cytokine expression, neutrophil infiltration and bacterial clearance in response to urinary tract infection in diabetic mice. Pathog Dis 73:ftv002. doi:10.1093/femspd/ftv002 25663347 PMC4443837

[16] Schwartz L, Salamon K, Simoni A, Eichler T, Jackson AR, Murtha M, Becknell B, Kauffman A, Linn-Peirano S, Holdsworth N, Tyagi V, Tang H, Rust S, Cortado H, Zabbarova I, Kanai A, Spencer JD. 2024. Insulin receptor signaling engages bladder urothelial defenses that limit urinary tract infection. Cell Rep 43:114007. doi:10.1016/j.celrep.2024.114007 38517889 PMC11094371

[17] Schwartz L, Simoni A, Yan P, Salamon K, Turkoglu A, Vasquez Martinez G, Zepeda-Orozco D, Eichler T, Wang X, Spencer JD. 2024. Insulin receptor orchestrates kidney antibacterial defenses. Proc Natl Acad Sci USA 121. doi:10.1073/pnas.2400666121 PMC11260129 38976738

[18] Murtha MJ, Eichler T, Bender K, Metheny J, Li B, Schwaderer AL, Mosquera C, James C, Schwartz L, Becknell B, Spencer JD. 2018. Insulin receptor signaling regulates renal collecting duct and intercalated cell antibacterial defenses. J Clin Invest 128:5634–5646. doi:10.1172/JCI98595 30418175 PMC6264632

[19] Saenkham P, Jennings-Gee J, Hanson B, Kock ND, Adams LG, Subashchandrabose S. 2020. Hyperglucosuria induced by dapagliflozin augments bacterial colonization in the murine urinary tract. Diabetes Obes Metab 22:1548–1555. doi:10.1111/dom.14064 32314507 PMC7571118

[20] Paudel S, Bagale K, Patel S, Kooyers NJ, Kulkarni R. 2021. Human urine alters methicillin-resistant Staphylococcus aureus virulence and transcriptome. Appl Environ Microbiol 87:e0074421. doi:10.1128/AEM.00744-21 34105987 PMC8315183

[21] Islam MJ, Bagale K, John PP, Kurtz Z, Kulkarni R. 2022. Glycosuria alters uropathogenic Escherichia coli global gene expression and virulence. mSphere 7:e0000422. doi:10.1128/msphere.00004-22 35477301 PMC9241551

[22] John PP, Baker BC, Paudel S, Nassour L, Cagle H, Kulkarni R. 2021. Exposure to moderate glycosuria induces virulence of group B streptococcus. J Infect Dis 223:843–847. doi:10.1093/infdis/jiaa443 32702082

[23] Haas B, Eckstein N, Pfeifer V, Mayer P, Hass MDS. 2014. Efficacy, safety and regulatory status of SGLT2 inhibitors: focus on canagliflozin. Nutr & Diabetes 4:e143–e143. doi:10.1038/nutd.2014.40 PMC4259905 25365416

[24] Vallon V, Platt KA, Cunard R, Schroth J, Whaley J, Thomson SC, Koepsell H, Rieg T. 2011. SGLT2 mediates glucose reabsorption in the early proximal tubule. J Am Soc Nephrol 22:104–112. doi:10.1681/ASN.2010030246 20616166 PMC3014039

[25] Xu B, Li S, Kang B, Zhou J. 2022. The current role of sodium-glucose cotransporter 2 inhibitors in type 2 diabetes mellitus management. Cardiovasc Diabetol 21:83. doi:10.1186/s12933-022-01512-w 35614469 PMC9134641

[26] Heerspink HJL, Jongs N, Chertow GM, Langkilde AM, McMurray JJV, Correa-Rotter R, Rossing P, Sjöström CD, Stefansson BV, Toto RD, Wheeler DC, Greene T, DAPA-CKD Trial Committees and Investigators. 2021. Effect of dapagliflozin on the rate of decline in kidney function in patients with chronic kidney disease with and without type 2 diabetes: a prespecified analysis from the DAPA-CKD trial. Lancet Diabetes Endocrinol 9:743–754. doi:10.1016/S2213-8587(21)00242-4 34619108

[27] Uitrakul S, Aksonnam K, Srivichai P, Wicheannarat S, Incomenoy S. 2022. The incidence and risk factors of urinary tract infection in patients with type 2 diabetes mellitus using SGLT2 inhibitors: a real-world observational study. Medicines (Basel) 9:59. doi:10.3390/medicines9120059 36547992 PMC9785475

[28] Nicolle LE, Capuano G, Ways K, Usiskin K. 2012. Effect of canagliflozin, a sodium glucose co-transporter 2 (SGLT2) inhibitor, on bacteriuria and urinary tract infection in subjects with type 2 diabetes enrolled in a 12-week, phase 2 study. Curr Med Res Opin 28:1167–1171. doi:10.1185/03007995.2012.689956 22548646

[29] Schneeberger C, Kazemier BM, Geerlings SE. 2014. Asymptomatic bacteriuria and urinary tract infections in special patient groups: women with diabetes mellitus and pregnant women. Curr Opin Infect Dis 27:108–114. doi:10.1097/QCO.0000000000000028 24296584

[30] Lega IC, Bronskill SE, Campitelli MA, Guan J, Stall NM, Lam K, McCarthy LM, Gruneir A, Rochon PA. 2019. Sodium glucose cotransporter 2 inhibitors and risk of genital mycotic and urinary tract infection: a population-based study of older women and men with diabetes. Diabetes Obes Metab 21:2394–2404. doi:10.1111/dom.13820 31264755

[31] Dave CV, Schneeweiss S, Kim D, Fralick M, Tong A, Patorno E. 2019. Sodium-glucose cotransporter-2 inhibitors and the risk for severe urinary tract infections: a population-based cohort study. Ann Intern Med 171:248–256. doi:10.7326/M18-3136 31357213 PMC6989379

[32] Liu J, Li L, Li S, Jia P, Deng K, Chen W, Sun X. 2017. Effects of SGLT2 inhibitors on UTIs and genital infections in type 2 diabetes mellitus: a systematic review and meta-analysis. Sci Rep 7:2824. doi:10.1038/s41598-017-02733-w 28588220 PMC5460243

[33] Fralick M, MacFadden DR. 2020. A hypothesis for why sodium glucose co-transporter 2 inhibitors have been found to cause genital infection, but not urinary tract infection. Diabetes Obes Metab 22:755–758. doi:10.1111/dom.13959 31943733

[34] Suzuki M, Hiramatsu M, Fukazawa M, Matsumoto M, Honda K, Suzuki Y, Kawabe Y. 2014. Effect of SGLT2 inhibitors in a murine model of urinary tract infection with Candida albicans. Diabetes Obes Metab 16:622–627. doi:10.1111/dom.12259 24400675

[35] Salamon K, Linn-Peirano S, Simoni A, de Dios Ruiz-Rosado J, Becknell B, John P, Schwartz L, Spencer JD. 2025. Analysing the influence of dapagliflozin on urinary tract infection vulnerability and kidney injury in mice infected with uropathogenic Escherichia coli. Diabetes Obes Metab 27:40–53. doi:10.1111/dom.15981 PMC11620950 39344841

[36] Mulvey MA, Schilling JD, Hultgren SJ. 2001. Establishment of a persistent Escherichia coli reservoir during the acute phase of a bladder infection. Infect Immun 69:4572–4579. doi:10.1128/IAI.69.7.4572-4579.2001 11402001 PMC98534

[37] Datsenko KA, Wanner BL. 2000. One-step inactivation of chromosomal genes in Escherichia coli K-12 using PCR products. Proc Natl Acad Sci USA 97:6640–6645. doi:10.1073/pnas.120163297 10829079 PMC18686

[38] George I, Kalairaj MS, Zimmern PE, Ware TH, Subashchandrabose S. 2024. Competitive fitness of asymptomatic bacteriuria E. coli strain 83972 against uropathogens in human urine. Infect Immun 92:e0017324. doi:10.1128/iai.00173-24 38780216 PMC11237815

[39] Saenkham P, Ritter M, Donati GL, Subashchandrabose S. 2020. Copper primes adaptation of uropathogenic Escherichia coli to superoxide stress by activating superoxide dismutases. PLoS Pathog 16:e1008856. doi:10.1371/journal.ppat.1008856 32845936 PMC7478841

[40] Subashchandrabose S, Hazen TH, Brumbaugh AR, Himpsl SD, Smith SN, Ernst RD, Rasko DA, Mobley HLT. 2014. Host-specific induction of Escherichia coli fitness genes during human urinary tract infection. Proc Natl Acad Sci USA 111:18327–18332. doi:10.1073/pnas.1415959112 25489107 PMC4280598

[41] Subashchandrabose S, Smith SN, Spurbeck RR, Kole MM, Mobley HLT. 2013. Genome-wide detection of fitness genes in uropathogenic Escherichia coli during systemic infection. PLoS Pathog 9:e1003788. doi:10.1371/journal.ppat.1003788 24339777 PMC3855560

[42] Hanson BS, Hailemariam A, Yang Y, Mohamed F, Donati GL, Baker D, Sacchettini J, Cai JJ, Subashchandrabose S. 2024. Identification of a copper-responsive small molecule inhibitor of uropathogenic Escherichia coli J Bacteriol 206:e0011224. doi:10.1128/jb.00112-24 38856220 PMC11270900

[43] King AJF. 2012. The use of animal models in diabetes research. Br J Pharmacol 166:877–894. doi:10.1111/j.1476-5381.2012.01911.x 22352879 PMC3417415

[44] Chakraborty G, Thumpayil S, Lafontant DE, Woubneh W, Toney JH. 2009. Age dependence of glucose tolerance in adult KK-Ay mice, a model of non-insulin dependent diabetes mellitus. Lab Anim (NY) 38:364–368. doi:10.1038/laban1109-364 19847180

[45] Powell DR, DaCosta CM, Smith M, Doree D, Harris A, Buhring L, Heydorn W, Nouraldeen A, Xiong W, Yalamanchili P, Mseeh F, Wilson A, Shadoan M, Zambrowicz B, Ding ZM. 2014. Effect of LX4211 on glucose homeostasis and body composition in preclinical models. J Pharmacol Exp Ther 350:232–242. doi:10.1124/jpet.114.214304 24849925

[46] Iwatsuka H, Shino A, Suzuoki Z. 1970. General survey of diabetic features of yellow KK mice. Endocrinol Jpn 17:23–35. doi:10.1507/endocrj1954.17.23 5468422

[47] Chen H, Zhang L, Hill WG, Yu W. 2017. Evaluating the voiding spot assay in mice: a simple method with complex environmental interactions. Am J Physiol Renal Physiol 313:F1274–F1280. doi:10.1152/ajprenal.00318.2017 28835420 PMC5814640

[48] Robinson CK, Saenkham-Huntsinger P, Hanson BS, Adams LG, Subashchandrabose S. 2022. Vaginal inoculation of uropathogenic Escherichia coli during estrus leads to genital and renal colonization. Infect Immun 90:e0053221. doi:10.1128/iai.00532-21 35357220 PMC9022555

[49] Smith SN, Hagan EC, Lane MC, Mobley HLT. 2010. Dissemination and systemic colonization of uropathogenic Escherichia coli in a murine model of bacteremia. mbio 1. doi:10.1128/mBio.00262-10 PMC2993011 21116344

[50] Jackson LA, Benson P, Neuzil KM, Grandjean M, Marino JL. 2005. Burden of community-onset Escherichia coli bacteremia in seniors. J Infect Dis 191:1523–1529. doi:10.1086/429344 15809912

[51] Alteri CJ, Smith SN, Mobley HLT. 2009. Fitness of Escherichia coli during urinary tract infection requires gluconeogenesis and the TCA cycle. PLoS Pathog 5:e1000448. doi:10.1371/journal.ppat.1000448 19478872 PMC2680622

[52] Foxman B. 2010. The epidemiology of urinary tract infection. Nat Rev Urol 7:653–660. doi:10.1038/nrurol.2010.190 21139641

[53] Brown JS, Wessells H, Chancellor MB, Howards SS, Stamm WE, Stapleton AE, Steers WD, Van Den Eeden SK, McVary KT. 2005. Urologic complications of diabetes. Diabetes Care 28:177–185. doi:10.2337/diacare.28.1.177 15616253

[54] Ozer A, Altuntas CZ, Izgi K, Bicer F, Hultgren SJ, Liu G, Daneshgari F. 2015. Advanced glycation end products facilitate bacterial adherence in urinary tract infection in diabetic mice. Pathog Dis 73. doi:10.1093/femspd/ftu004 PMC4444075 25986378

[55] Alteri CJ, Himpsl SD, Shea AE, Mobley HLT. 2019. Flexible metabolism and suppression of latent enzymes are important for Escherichia coli adaptation to diverse environments within the host. J Bacteriol 201:e00181-19. doi:10.1128/JB.00181-19 31160397 PMC6657593

[56] Sendtner GW, Miranda J, Naumann P, Weiss M, Güls P, Molitor E, Scheidt U, Schmidt A, Ludwig KU, Hilger A, Dobrindt U, Mayrhofer T, Kurts C, Wagenlehner F, Shevshuk O, von Vietinghoff S. 2026. Sodium glucose transporter 2 inhibition maintains kidney antibacterial response by decreasing complement C1q. Kidney Int:S0085-2538(26)00009-8. doi:10.1016/j.kint.2026.01.003 41565025

[57] Deltourbe L, Lacerda Mariano L, Hreha TN, Hunstad DA, Ingersoll MA. 2022. The impact of biological sex on diseases of the urinary tract. Mucosal Immunol 15:857–866. doi:10.1038/s41385-022-00549-0 35869147 PMC9305688

[58] Puckrin R, Saltiel MP, Reynier P, Azoulay L, Yu OHY, Filion KB. 2018. SGLT-2 inhibitors and the risk of infections: a systematic review and meta-analysis of randomized controlled trials. Acta Diabetol 55:503–514. doi:10.1007/s00592-018-1116-0 29484489

