# Supplementary material for: Diabetes and hyperglucosuria exacerbate the severity of urinary tract infection caused by uropathogenic E. coli in the mouse model
Source: Infect Immun. 2026 Apr 30;94(6):e00172-26. doi: 10.1128/iai.00172-26 (PMC13248725; doi:10.1128/iai.00172-26)
Supplement: Supplemental material — Fig. S1 to S8. [file iai.00172-26-s0001.pdf]

SUPPLEMENTAL FIGURES

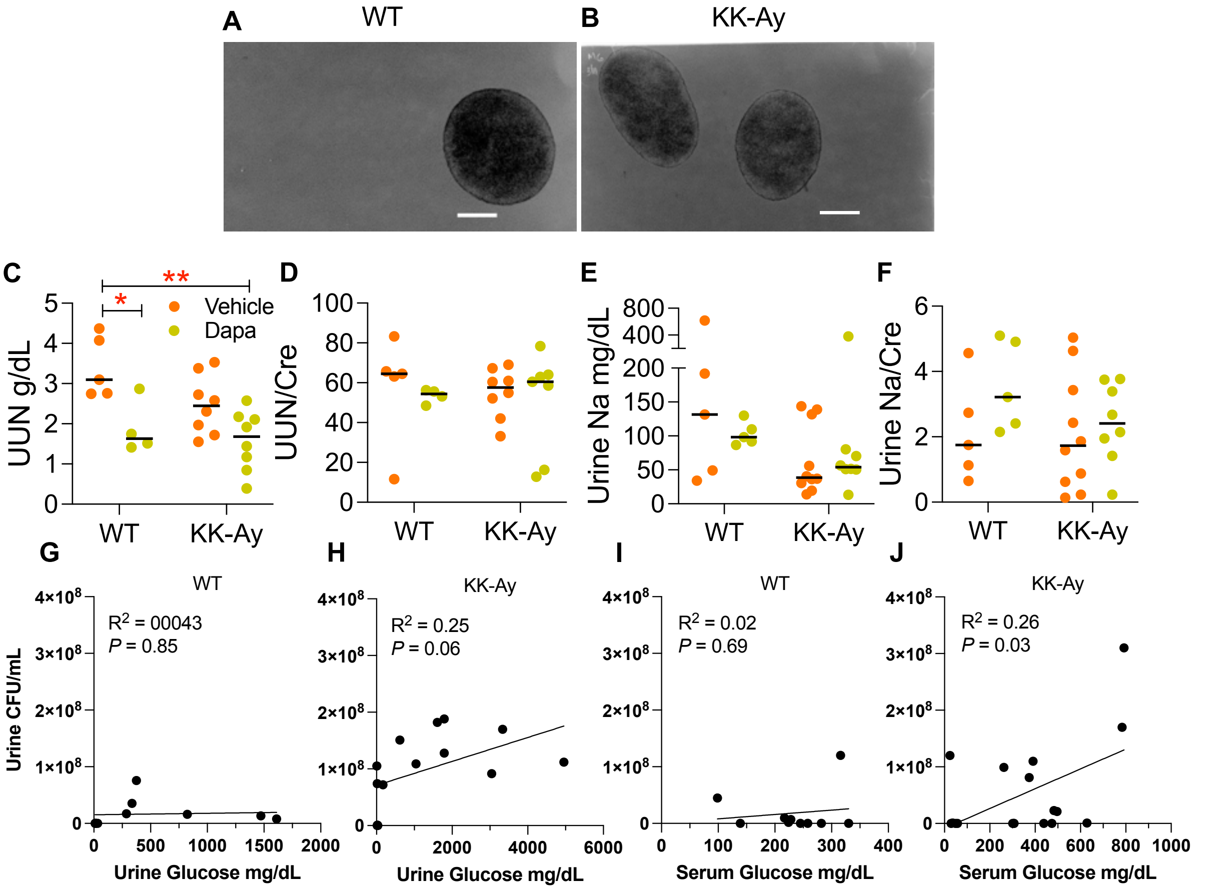

Figure S1. Urine voiding pattern, urine analytes and correlation between urine UPEC load and urine and serum glucose levels. A representative image from urine void spot assays from diabetic and healthy WT littermates (A&B). Scale bar = 1 cm. Absolute and normalized urine urea nitrogen (UUN) and sodium (Na) levels in diabetic and control mice. Each symbol represents a mouse and bars represent median. Scatter plot showing correlation between urine glucose (G&H) and serum glucose (I&J) and urine UPEC load (CFU/mL) with linear regression, coefficient of determination (R^2) and P value.

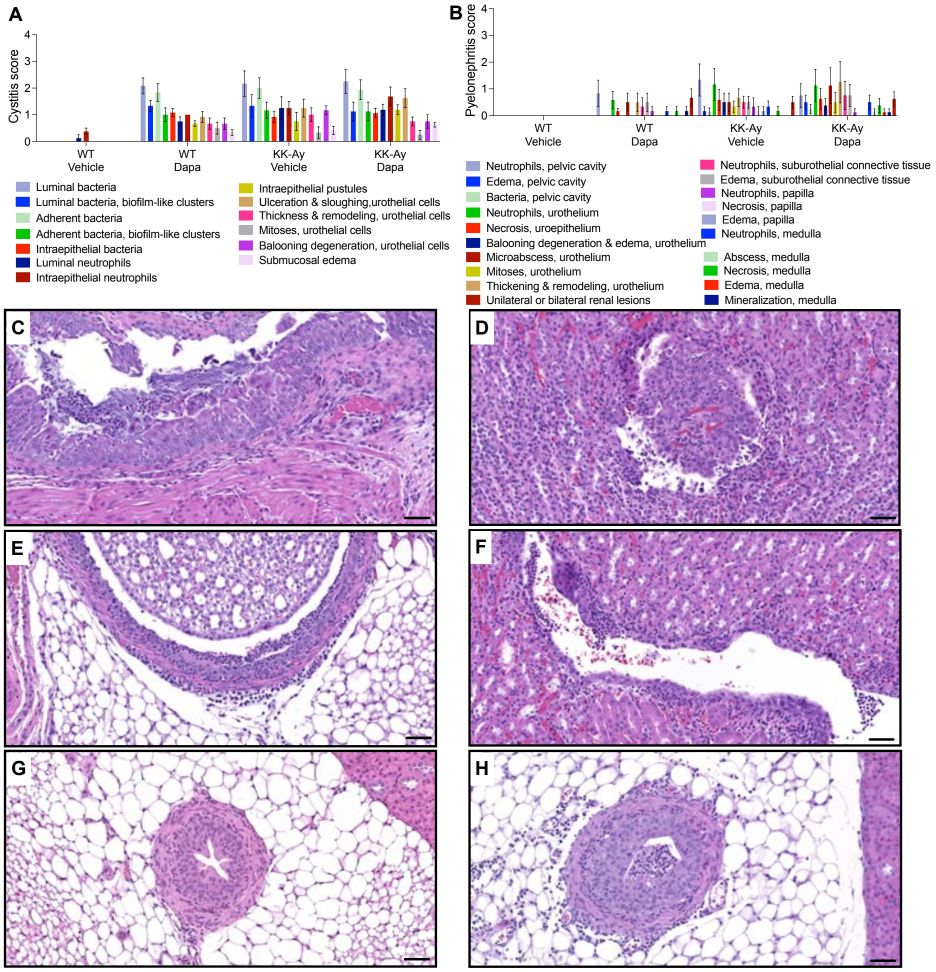

Figure S2. Higher degree of inflammation and tissue injury in dapagliflozin-treated diabetic mice. A score (0-4) was assigned for urinary bladder (A) and kidneys (B) based on indicated criteria. (C) Neutrophilic cystitis with ulceration and erosion of mucosa, and numerous bacterial biofilms. (D) Early abscess in renal medulla. (E) Neutrophilic pyelitis, and neutrophilic inflammation of suburothelial connective tissue. (F) Numerous microabscess with necrosis of pelvic urothelium. (G) Normal ureter. (H) Neutrophilic ureteritis and periureteritis. Mean + SEM. KK-Ay, diabetic mice; WT, healthy littermates; Dapa, dapagliflozin; and Vehicle, 0.5% w/v carboxymethylcellulose in water. Scale bar = 50 µm.

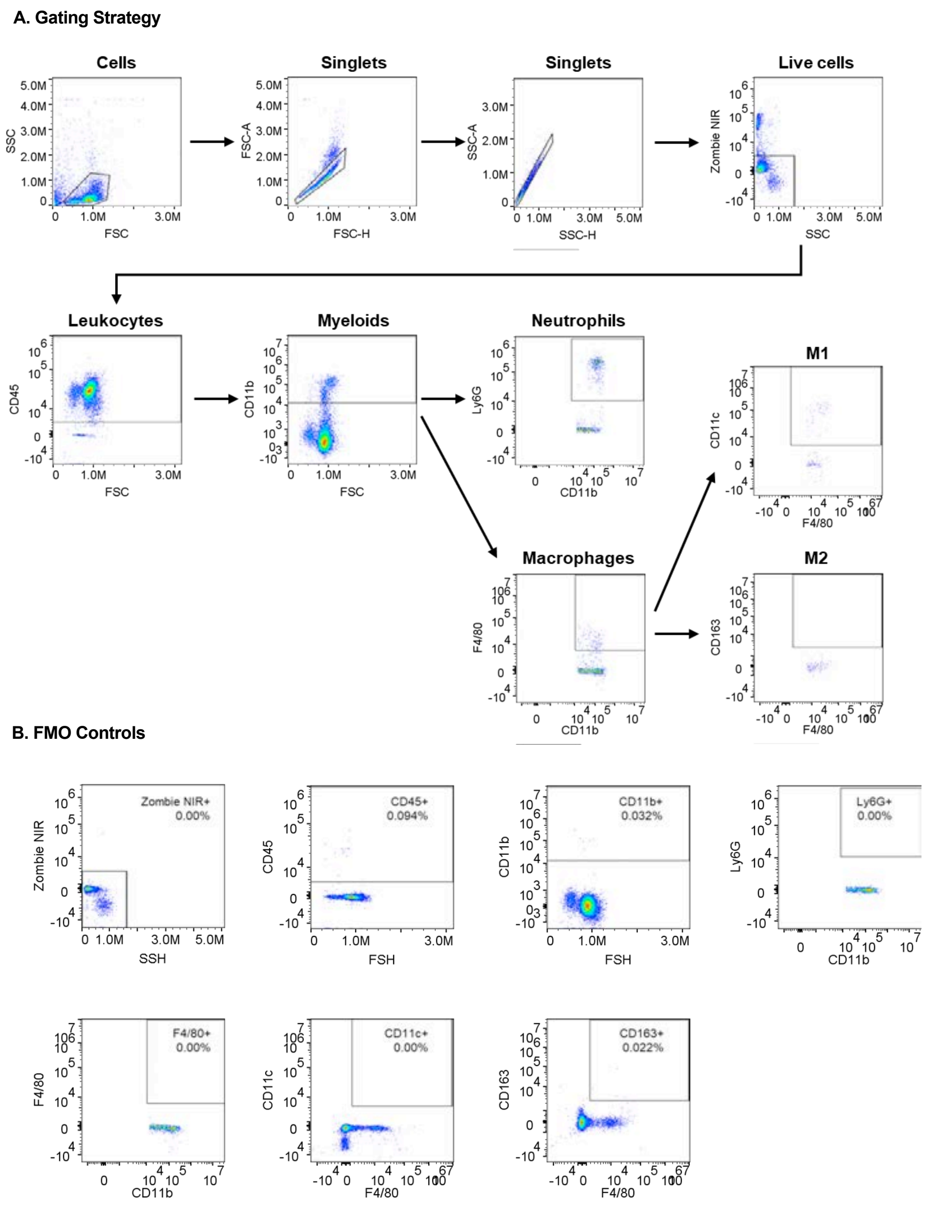

Figure S3. Gating strategy and fluorescence minus one controls (FMO) used in flow cytometry. Neutrophil, and macrophages infiltration during acute UTI was quantified by flow cytometry from single-cell suspension of urinary bladder cells using gating strategy (A), and FMO controls (B). FMO controls were used for accurate gate placement for each cell surface marker and Zombie live/dead stain.

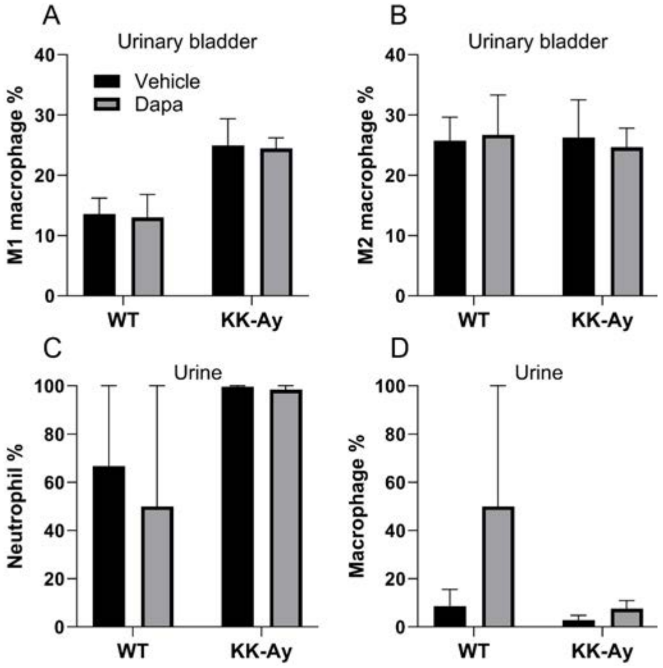

Figure S4. M1 and M2 macrophage infiltration in the urinary bladder during UTI. Diabetic KK-Ay and healthy WT mice (n = 3/group) were inoculated with UPEC UTI89 transurethrally. The urinary bladders, and urine were analyzed by flow cytometry for M1 and M2 macrophages as a percentage of total macrophage counts in urinary bladder (A and B), and neutrophils and macrophages as a percentage of total myeloid cells in urine (C and D). Mean + SEM.

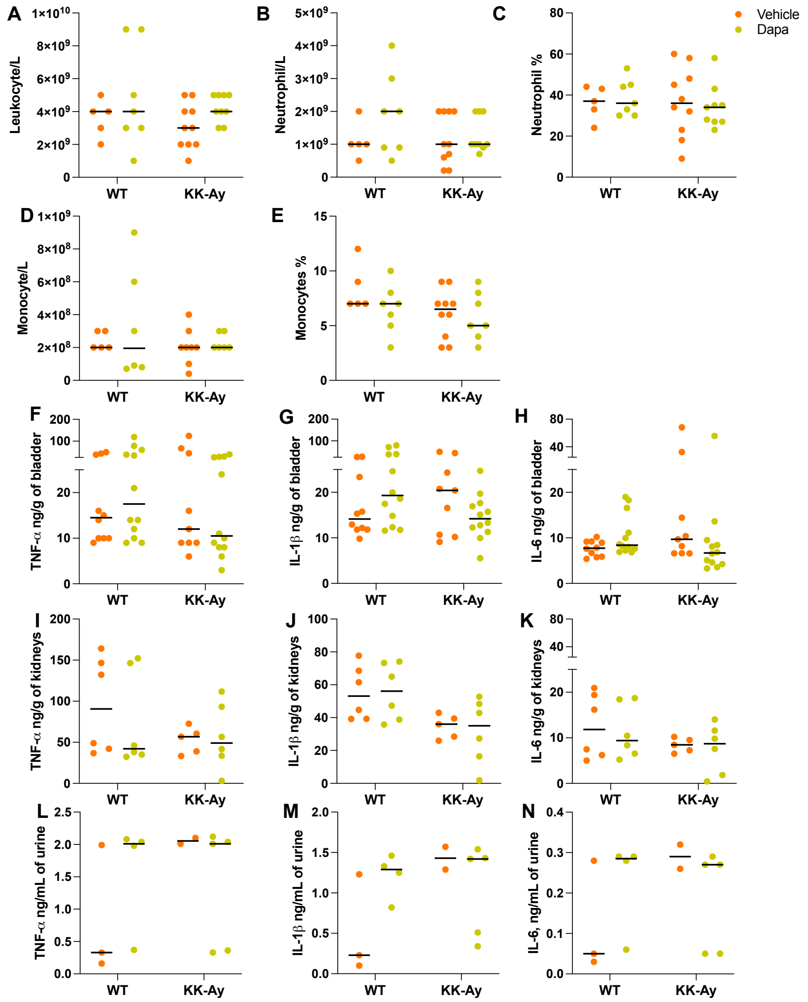

Figure S5. Similar CBC and proinflammatory cytokines levels in diabetic and healthy mice treated with dapagliflozin and vehicle during acute UTI. Blood was collected via cardiac puncture after euthanasia post-infection from female diabetic KK-Ay mice (n = 5-10/group), and healthy, WT littermates mice (n = 5-10/group). Complete blood count (CBC) was performed and leukocytes (A), neutrophils (B and C), monocytes (D, and E) count were determined. Urine, urinary bladders, and kidneys were collected at day 2 of infection from mice. Urine and organ homogenates were analyzed by enzyme-linked immunosorbent assay. TNF-α, IL-1β, IL-6 quantification in ng/g of bladder (F-H), kidneys (I-K), and pg/mL of urine (L-N) are depicted. Each symbol represents a mouse, and bar indicated median.

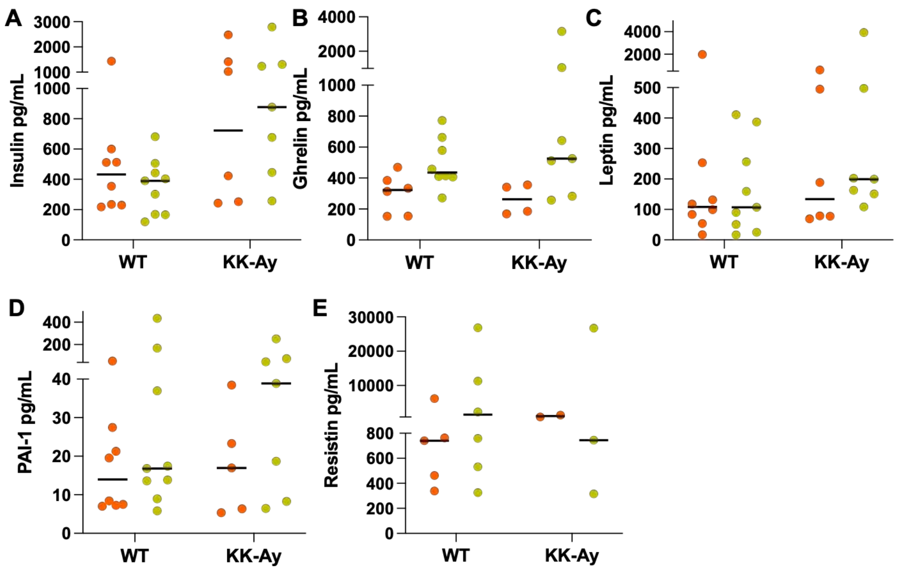

Figure S6. Biomarkers for diabetes. Female diabetic KK-Ay mice (n = 6-8/group), and female, healthy, WT controls (n= 6-8/group) were randomly assigned to dapagliflozin and vehicle groups and inoculated with UPEC strain UTI89 transurethrally. Blood was collected immediately after euthanasia by cardiac puncture. Insulin (A), ghrelin (B), leptin (C), plasminogen activator inhibitor-1 (PAI-1) (D), and resistin (E) levels in serum were quantified using Pro Mouse Diabetes 8-Plex BioRad Assay. Each symbol represents a mouse, and bars indicate median. Dapa, dapagliflozin; Vehicle, 0.5% carboxymethylcellulose in sterile water.

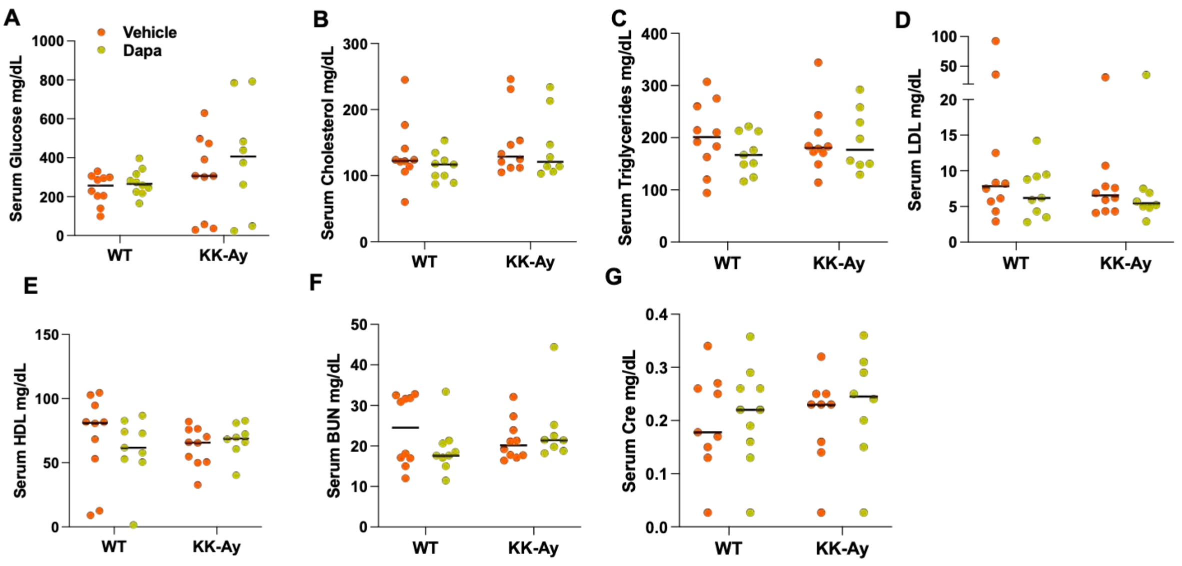

Figure S7. Serum glucose and lipid metabolism markers in diabetic mice compared to healthy mice. Female diabetic KK-Ay mice (n = 5-10/group), and female, healthy, WT controls (n= 5-10/group) were inoculated with UPEC strain UTI89 transurethrally. Blood was collected immediately after euthanasia by cardiac puncture, and serum was collected after centrifugation. Glucose (A), cholesterol (B), triglycerides (C), HDL (D), LDL (E), BUN (F), and creatinine (G) levels in serum were quantified. Each symbol represents a mouse, and bars indicate median. Dapa, dapagliflozin; Vehicle, 0.5% carboxymethylcellulose in sterile water.

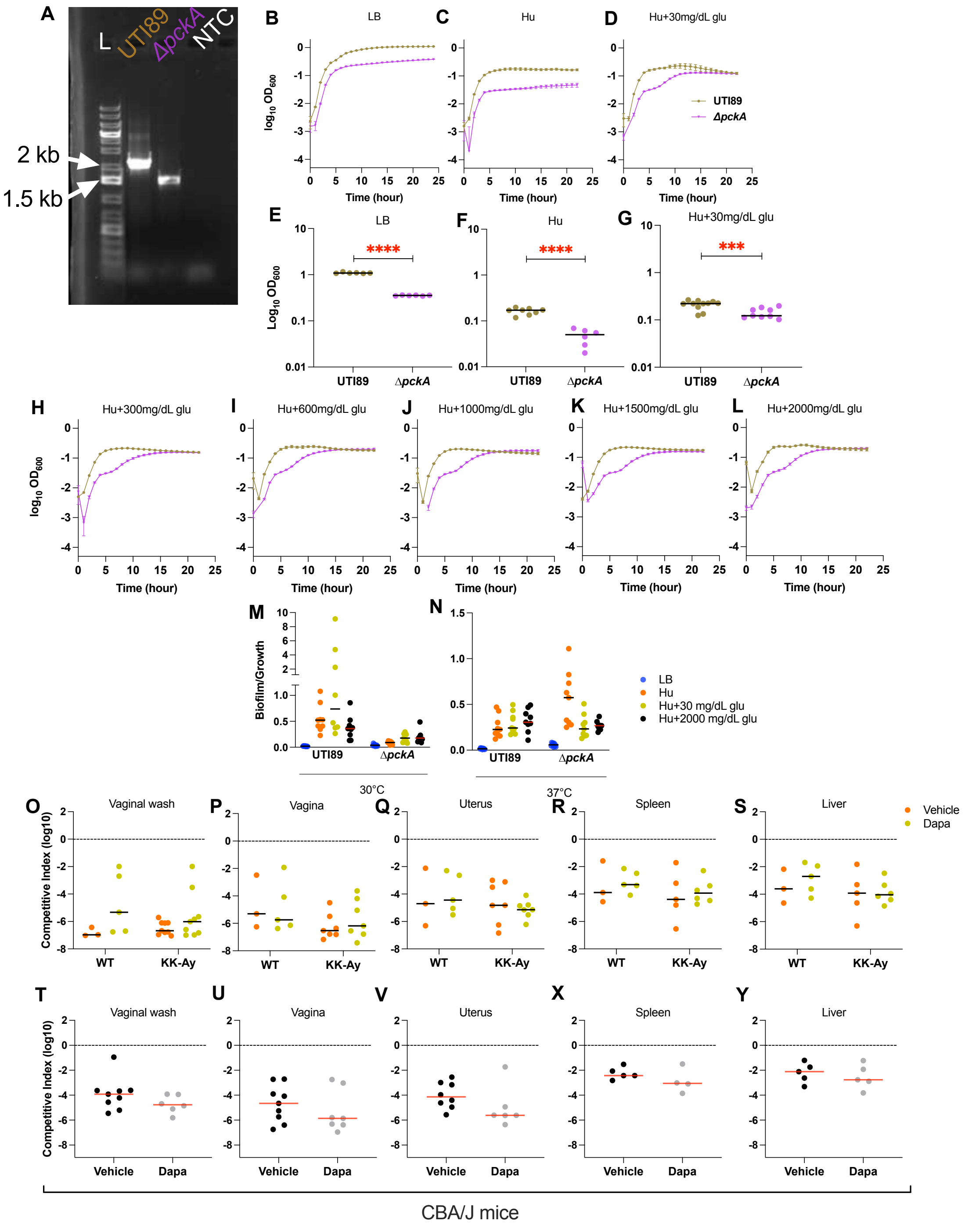

Figure S8. Gluconeogenesis is required for UPEC fitness in the genital tract and systemic sites. UTI89Δ*pckA* was constructed by lambda recombineering method and verified by PCR and gel-electrophoresis (A). L, 1kp plus DNA ladder; UTI89; UTI89Δ*pckA*; and NTC, no template control. Growth kinetics was determined in LB and human urine with or without glucose (glu) at

indicated levels (B-D&H-L). Cell densities of stationary phase cultures in LB and urine were compared (E-G). Biofilm biomass (OD_{550}) normalized to growth (OD_{600}) at 30ºC (M), and 37ºC (N) is depicted. Bars represent mean (N = 9). Female diabetic KK-Ay, healthy WT littermates, and non-diabetic (CBA/J) mice were co-infected with UPEC strain UTI89 and UTI89Δ*pckA* (1:1) transurethrally. Mice were euthanized at 48 hours post-infection, and competitive indices were calculated in vaginal lavage and indicated organs of diabetic mice and littermate controls (O-S), and in the non-diabetic CBA/J mice (T-Y). Dotted line, no loss of fitness in mutant relative to parental wild-type strain (competitive index = 1). Each symbol represents a mouse, and bars indicate median. Glu, glucose; Dapa, dapagliflozin; and Vehicle, 0.5% carboxymethylcellulose in sterile water.
